# Sulforaphane potentiates anticancer effects of doxorubicin and attenuates its cardiotoxicity in a breast cancer model

**DOI:** 10.1371/journal.pone.0193918

**Published:** 2018-03-08

**Authors:** Chhanda Bose, Sanjay Awasthi, Rajendra Sharma, Helen Beneš, Martin Hauer-Jensen, Marjan Boerma, Sharda P. Singh

**Affiliations:** 1 University of Arkansas for Medical Sciences, Department of Geriatrics, Little Rock, Arkansas, United States of America; 2 Texas Tech Health Sciences Center, Division of Hematology & Oncology, Department of Internal Medicine, Lubbock, Texas, United States of America; 3 University of Arkansas for Medical Sciences, Department of Pharmacology and Toxicology, Little Rock, Arkansas, United States of America; 4 University of Arkansas for Medical Sciences, Department of Neurobiology and Developmental Sciences, Little Rock, Arkansas, United States of America; 5 University of Arkansas for Medical Sciences, Division of Radiation Health, Little Rock, Arkansas, United States of America; 6 Central Arkansas Veterans Healthcare System, Little Rock, Arkansas, United States of America; National Institutes of Health, UNITED STATES

## Abstract

Breast cancer is the most common malignancy in women of the Western world. Doxorubicin (DOX) continues to be used extensively to treat early-stage or node-positive breast cancer, human epidermal growth factor receptor-2 (HER2)-positive breast cancer, and metastatic disease. We have previously demonstrated in a mouse model that sulforaphane (SFN), an isothiocyanate isolated from cruciferous vegetables, protects the heart from DOX-induced toxicity and damage. However, the effects of SFN on the chemotherapeutic efficacy of DOX in breast cancer are not known. Present studies were designed to investigate whether SFN alters the effects of DOX on breast cancer regression while also acting as a cardioprotective agent. Studies on rat neonatal cardiomyocytes and multiple rat and human breast cancer cell lines revealed that SFN protects cardiac cells but not cancer cells from DOX toxicity. Results of studies in a rat orthotopic breast cancer model indicated that SFN enhanced the efficacy of DOX in regression of tumor growth, and that the DOX dosage required to treat the tumor could be reduced when SFN was administered concomitantly. Additionally, SFN enhanced mitochondrial respiration in the hearts of DOX-treated rats and reduced cardiac oxidative stress caused by DOX, as evidenced by the inhibition of lipid peroxidation, the activation of NF-E2-related factor 2 (Nrf2) and associated antioxidant enzymes. These studies indicate that SFN not only acts synergistically with DOX in cancer regression, but also protects the heart from DOX toxicity through Nrf2 activation and protection of mitochondrial integrity and functions.

## Introduction

Breast cancer is the most common malignancy in western women. Doxorubicin (DOX) is used to treat early-stage or node-positive breast cancer, human epidermal growth factor receptor-2 (HER2)-positive breast cancer, and metastatic disease. However, one of the major side effects of DOX treatment is cardiotoxicity [1–3], which significantly contributes to morbidity and mortality in breast cancer patients treated with this drug. The occurrence of cardiac events associated with DOX therapy in cancer patients is estimated between 10% to 25%, and the prevalence of left ventricular contractile dysfunction in patients with a cumulative DOX dose of approximately 430–600 mg/m^2^ is about 50–60% of the patients with cardiomyopathic episodes [4, 5]. Recent advancements in cancer therapies have increased survivorship; however, cardiotoxic side effects remain clinically challenging [6]. Recently there have been reports of strategies to reduce the occurrence and severity of DOX-induced cardiotoxicity, studies have demonstrated that these pharmaceuticals have their own side effects [7–15].

The mechanism for the anti-cancer action of DOX seems to be distinct from the mechanisms responsible for its cardiotoxicity. DOX anti-cancer activity is based on producing DNA damage and inhibiting cell replication [3, 16, 17]. These effects are less relevant for non-replicating cardiomyocytes. Instead, the primary cause of cardiotoxicity appears to be DOX-induced oxidative/electrophilic stress and mitochondrial dysfunction in the heart [18–22]. Exposure to DOX causes initial mitochondrial injury, which compromises cellular bioenergetics capacity, and initiates a delayed progression of cardiac damage [23, 24]. DOX-induced cardiac oxidative stress may result in part from its inhibition of expression and functions of the transcription factor Nrf2 (Nuclear Factor-E2-related factor 2) [17].

The KEAP1-Nrf2-ARE antioxidant system is a principal means by which cells respond to oxidative and xenobiotic stresses by activating antioxidant and anti-electrophile enzymes [25, 26]. In this system, the transcription factor Nrf2 binds to the antioxidant response element (ARE) of several target genes. Nrf2 activity is under the regulation of Kelch ECH associating protein 1 (KEAP1). Sulforaphane (SFN), a safe phytochemical and dietary supplement, enhances signaling by Nrf2 and consequent cytoprotective gene activation. SFN treatment has been shown to reduce oxidative stress and counter DOX cardiotoxicity in animal models [27, 28]. We previously demonstrated that SFN protects the heart from DOX-induced toxicity in the mouse as well as in cultured rat H9c2 cardiomyoblast cells [27].

SFN also inhibits carcinogenesis and metastases through various mechanisms including the activation of tumor suppressor genes, activation of caspase-3, induction of cell cycle arrest, reduction of estrogen receptor alpha (ERα) levels, and inhibition of DNA methyltransferase (DNMT) and telomerase reverse transcriptase (TERT) [29–32]. Therefore, SFN is currently being tested in several clinical trials of cancer treatment (https://clinicaltrials.gov/ct2/results?term=sulforaphane&pg=1) [33–37]. However, it has not yet been exploited as an adjuvant to existing cancer treatment regimens. We hypothesized that SFN co-treatment might not only protect the heart from the harmful effects of DOX, but also enhance the anticancer potential of DOX.

Therefore, the present studies were a pre-clinical evaluation of the efficacy of SFN as an adjuvant to DOX chemotherapy, and a comparison of its effects on Nrf2 activity in cancer cells and primary cardiomyocytes. Since the hearts of cancer patients are generally under oxidative and electrophilic stress generated by cancer therapy, and are likely also stressed because of the oxidative and inflammatory responses elicited by cancer [38–42], we also tested the effects of DOX and SFN in a rat breast cancer model. Since DOX has been shown to cause an elevation in the serum levels of cytokines TNF-α and IL-6 due to the disruption of the intestinal epithelium, enabling the leakage of endotoxin from gut microflora into the circulation [43, 44], and since SFN can attenuate inflammation [45, 46], we examined inflammatory cytokine levels in our rat model.

## Materials and methods

### Ethics statement

The work was performed in accordance with a protocol approved by the Central Arkansas Veterans Healthcare System (CAVHS) Institutional Animal Care and Use Committee (protocol # 443033). Animals were housed in the Veterinary Medical Unit at the Central Arkansas Veterans Healthcare System in Little Rock, Arkansas. All procedures and experiments complied with the guidelines to minimize animal suffering. Once a week, tumor size was measured by ultrasound as described below. If the animals became moribund, if pain was detected during the study, or tumor-bearing rats developed tumors larger than 15–20 mm^3^, the animals were euthanized immediately using CO_2_ inhalation.

### Cardiac and cancer cell culture

Neonatal rat cardiac ventricular myocytes (NRCM) were isolated by standard techniques from Sprague-Dawley rats, as previously described [47, 48]. Briefly, 2–3 day old rat pups were decapitated, heart ventricles aseptically dissected, minced, and exposed to trypsin treatment overnight at 4°C. Subsequently, the cells were dissociated by repetitive collagenase treatment at 37°C, centrifuged and rinsed with Hanks’ Balanced Salt solution (HBSS) kept at 4°C. Cells were preplated in chemically defined DMEM supplemented with 10% fetal bovine serum (FBS) (Thermo Fisher Scientific, Waltham, MA) and 30% penicillin/streptomycin for 20–30 min at 37°C. Subsequently, the non-attached cells (myocytes) were counted and plated at standard seeding density concentrations. After 24 hrs, the medium was changed and cells were maintained in the same chemically defined medium. Typically, cells started spontaneous beating after 2 d in culture, at which time the experiments were initiated.

Four breast cancer cell lines, obtained from the American Type Culture Collection (ATCC, Manassas, VA), were used for *in vitro* studies: MCF 10A (ATCC CRL-10317; noninvasive cells) and three invasive lines with increasingly aggressive profiles, MCF-7 (ATCC HTB-22), 13762 MAT B III (MAT B III, ATCC CRL-1666) and MDA-MB-231 (ATCC HTB-26, an invasive, human triple-negative breast cancer (TNBC) cell line). TNBC is a highly aggressive clinical subtype of breast cancer characterized by a lack of ER, progesterone receptor (PR) and HER2 expression [49]. MCF-10A cells were grown in Mammary Epithelial Cell Growth Medium (MEGM) (Thermo Fisher Scientific) supplemented with Single Quots and cholera toxin (Lonza, Basel, Switzerland). MCF-7 cells were grown in Eagle's Minimum Essential Medium (MEM) (Thermo Fisher Scientific) containing 10% FBS and 0.01 mg/ml human recombinant insulin. MAT B III cells were grown in McCoy's 5a Medium supplemented with 10% FBS, and MDA-MB-231 cells were cultured in ATCC-formulated Leibovitz's L-15 Medium supplemented with 10% FBS. All cells were grown in a humidified 5% CO_2_ incubator at 37°C.

### Cytotoxicity assays

The cell survival of the NRCM, breast cancer cells (MAT B III, MCF-7 and MDA-MB-231) and normal breast epithelial cells (MCF-10A) was assayed following SFN (Sigma, St Louis MO) and/or DOX (Sigma) administration. In a pilot experiment, the IC_50_ of SFN and DOX was determined for each cancer cell line. 5,000 cells grown overnight in 96-well plates were exposed to 2.5 μM SFN (which had resulted in significant protection of H9c2 cells) [36] or vehicle for 12 hrs before exposure to 5 μg/ml DOX or vehicle (DMSO). After 24 hrs, cell viability was determined using calcein blue AM (Thermo Fisher Scientific) [50].

### Animal models and treatment plan

Female Sprague Dawley rats (age 6–8 weeks) were obtained from Charles River Laboratories (Hollister, CA) and housed in the Veterinary Medical Unit at the CAVHS in Little Rock, AR. First, non-tumor bearing rats were examined for the cardiac effects of SFN administration during chronic DOX treatment. A pilot study had been performed to establish the dose, frequency, and duration of SFN exposure, and a dose of 4 mg/kg of SFN for 5 days/week was found to be effective in cardioprotection. Hence, SFN (Sigma) at a dose of 4 mg/kg (or vehicle, PBS) was administered orally 5 days/week for 4 weeks. After one week of SFN (or vehicle) pretreatment, rats from each group were injected (*i*.*p*.) with DOX (Sigma) at a dose of 5 mg/kg once a week to a total of 20 mg/kg (similar to a dose of 120 mg/m^2^ for humans) or vehicle (PBS). The same dosing of SFN continued concomitantly. Each experimental group contained 8 animals.

**Fig 1:**
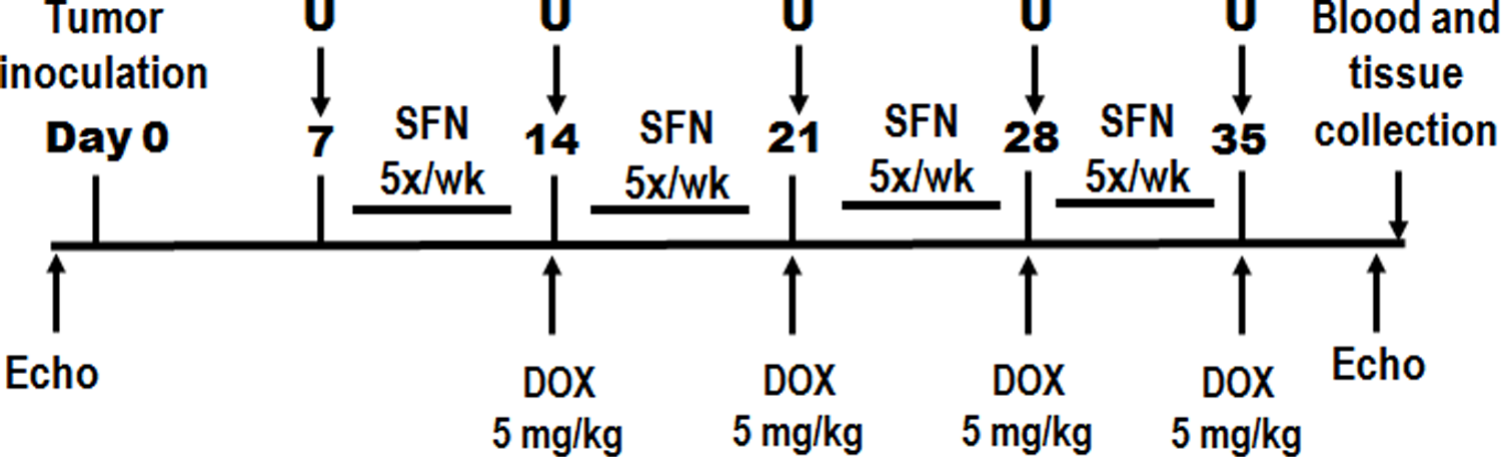
Treatment schedule in an orthotopic breast cancer model. Female Sprague-Dawley rats were inoculated with breast cancer cells (13762 MAT B III) to produce mammary tumors, and tumor size was monitored weekly by ultrasound. Following a 1-week pretreatment with SFN (4 mg/kg/day, 5 days per week), the rats received four doses of DOX (5 mg/kg) once a week to a total of 20 mg/kg, while the same dosing of SFN continued.

Second, an orthotopic breast cancer model was used to assess the effects of SFN on DOX-induced tumor regression and cardiac toxicity. For this purpose, *in vitro* cultures of the rat mammary gland cancer cell line MAT B III were re-suspended in PBS to a final concentration of 1x10^7^ cells/ml. Rats were anesthetized with 1–3% isoflurane followed by an injection of 1x10^6^ cells (100 μ03BCl) into one of the mammary glands. When tumors were ~5–6 mm^3^ in diameter, rats started receiving treatments. SFN was administered as described for the non-tumor bearing rats above. DOX was administered *i*.*p*. at a dose of 2.5 mg/kg or 5 mg/kg once a week to a total of 10 and 20 mg/kg, respectively. Treatment schedule and ultrasound measurements are indicated schematically in Fig 1. Animals were sacrificed on day one after the completion of DOX and SFN treatment, and heart and tumor samples collected for further analysis.

### Echocardiography

Cardiac function was assessed 8 days before the start of SFN treatment and on the day after completion of DOX and SFN treatment by echocardiography using the Vevo 770 high-resolution *in vivo* micro-imaging system (VisualSonics, Toronto, Canada) with an RMV 707B Scanhead (center frequency 17.5 MHz, frequency band 11.5–23.5 MHz, and focal length 17.5 mm), as we have reported previously [51].

### Measurement of breast tumor growth patterns

Once a week after tumor cell injection, tumor diameters were measured by high-resolution ultrasound using a VisualSonics Vevo 770 imaging system, and tumor volume were calculated according to the formula: V = (4/3) × π × (L/2) × (L/2) × (D/2), where L is tumor length and D is tumor depth [52, 53].

### High resolution respirometry

Immediately upon tissue collection, myocardial fibers isolated from the left ventricles [54, 55] were permeabilized and assayed for mitochondrial complex activities, using a substrate inhibitor titration protocol on an oxygraph (Oroboros Instruments, Innsbruck, Austria) [56]. Complex I activity was tested by addition of malate and glutamate to initiate respiration. To achieve maximal active respiration, ADP was added, followed by inhibition of complex I respiration by addition of rotenone. Complex II/III respiration was stimulated by addition of succinate followed by addition of the complex III inhibitor antimycin A. Complex IV respiration was measured by addition of N,N,N9,N9-tetramethyl-p-phenylenediamine (TMPD) in ascorbate; complex IV activity was inhibited by adding sodium azide. Respiratory rates were expressed per mg of dry weight.

### Transmission electron microscopy

Left ventricular heart biopsy samples from control and treated groups of animals were fixed overnight in 2.5% glutaraldehyde/0.05% malachite green in 0.1M sodium cacodylate buffer. Samples were post-stained with 1% osmium tetroxide/0.8% potassium hexacyanoferrate (III), 1% tannic acid, and 0.5% uranyl acetate followed by dehydration in a graded alcohol series and propylene oxide and embedded in Araldite-Embed 812 (Electron Microscopy Sciences, Hartfield, PA). 50-nm sections were collected on a Leica UC7 microtome and post-stained with uranyl acetate and lead citrate. Images were collected using a Tecnai F20 (FEI Company, Hillsboro, OR) transmission electron microscope at 80kv.

### Determination of oxidative stress

Levels of lipid peroxidation, reduced glutathione (GSH) and 4-hydroxynonenal (4-HNE) adducts were measured in the heart biopsy samples obtained from treated and vehicle-treated (control) groups of animals. Lipid peroxidation was determined by the reaction of malondialdehyde (MDA) with thiobarbituric acid (TBA) to form a colorimetric product, proportional to the MDA present in the tissue, as described previously [11]. Reduced GSH was determined by using a kit from BioVision (Milpitas, California). 4-HNE adducts were detected in tissue lysates as per our previously described method [27].

### Antioxidant and anti-electrophile enzyme activity

Superoxide dismutase (SOD), catalase, aldehyde dehydrogenase (ALDH) and aldo-keto reductase1 (AKR) enzyme activities were assayed in heart tissue homogenates using previously described methods [51].

### Inflammatory cytokine assay

Whole blood was collected two days after the third DOX injection by retro-orbital puncture in commercially available tubes from Becton Dickinson (BD). After collection of the whole blood, allowed the blood to clot by leaving it undisturbed at room temperature for 15–30 minutes. Serum was separated by centrifuging at 1,000–2,000 x g for 10 minutes in a refrigerated centrifuge. ELISAs were performed to assess serum IL-6 and TNF-α levels using kits from R&D Systems, Inc. (Minneapolis, MN).

### Active Nrf2 binding assay

Nuclei were extracted from heart ventricular tissue from control and treated groups of animals, and from NRCM and cancer cells (treated as described above) using the Nuclear Extract Kit (Active Motif, Carlsbad, CA) as per manufacturer’s instructions. Nrf2 activity was measured in nuclear extracts by an Nrf2-DNA-binding ELISA kit on nuclear fractions prepared from cardiac tissue and cells using Trans AM Nrf2 Kits (Active Motif) as we have reported before [51].

### HDAC, DNMT and ERα assays

The effect of SFN and/or DOX on HDAC and DNMT activities and detection of ER°α in breast cancer cells was determined in nuclear extracts. Briefly, MAT B III, MCF-7 and MDA-MB-231 cells were treated with SFN (2.5 μ03BCM) and/or DOX (5 µg/ml) for 48 hrs and only adherent cells harvested. Nuclear extracts were then prepared using a nuclear extraction kit (Active Motif) and assessed with the Epigenase HDAC activity assay kit (Epigentek, Farmingdale, NY), ERα ELISA kit (Abcam, Cambridge, MA) and DNMT Activity Quantification Kit (Abcam) according to the manufacturers’ instructions.

### Caspase-3 activation assay

Caspase-3 activity was assessed in breast cancer cells (MAT B III, MCF-7 and MDA-MB-231). Of each cell line, 10,000 cells were plated in white-walled 96-well plates compatible with a luminometer (Molecular Devices, Sunnyvale, CA). After 24 hrs, cells were treated with SFN (2.5 μM) and/or DOX (5 μg/ml) for 48 hrs with control cells receiving an equivalent volume of vehicle. Caspase-3 activity was measured using the Caspase-Glo^®^ 3/7 Assay Systems kit (Promega, Madison, WI) according to the manufacturer’s instructions [57].

### Data availability and statistical analysis

A raw dataset (an Excel file containing all pertinent raw data obtained from cell culture and animal experiments in this study) is provided as the Supporting Information file. Values are expressed as mean ± standard deviation (SD). Data were analyzed for significance by ANOVA with post-hoc Tukey-Kramer or Newman-Keuls test, and *p*-values less than 0.05 were considered significant.

## Results

### SFN protects against DOX-induced cardiomyopathy in non-tumor bearing rats

We first conducted a pilot study to determine survival of rats not carrying tumors and treated with different SFN and DOX dose combinations. We found that SFN (4 mg/kg, 5 days/week) protected against mortality and cardiac dysfunction induced by DOX (5 mg/kg x 4, total 20 mg/kg). Only 11% of the rats treated with DOX alone survived to the end of the 7-week experiment, compared to a 62% survival rate for the SFN+DOX treated animals. Comparison of SFN+DOX vs. DOX treatment alone showed a significant difference in mortality rates by a log-rank test (*p* = 0.0054). By Cox regression analysis SFN+DOX-treated rats exhibited a 85% reduction in hazard of dying from DOX exposure as compared to rats treated by DOX alone (S1 Fig). We therefore selected these SFN and DOX doses for the current study.

**Fig 2:**
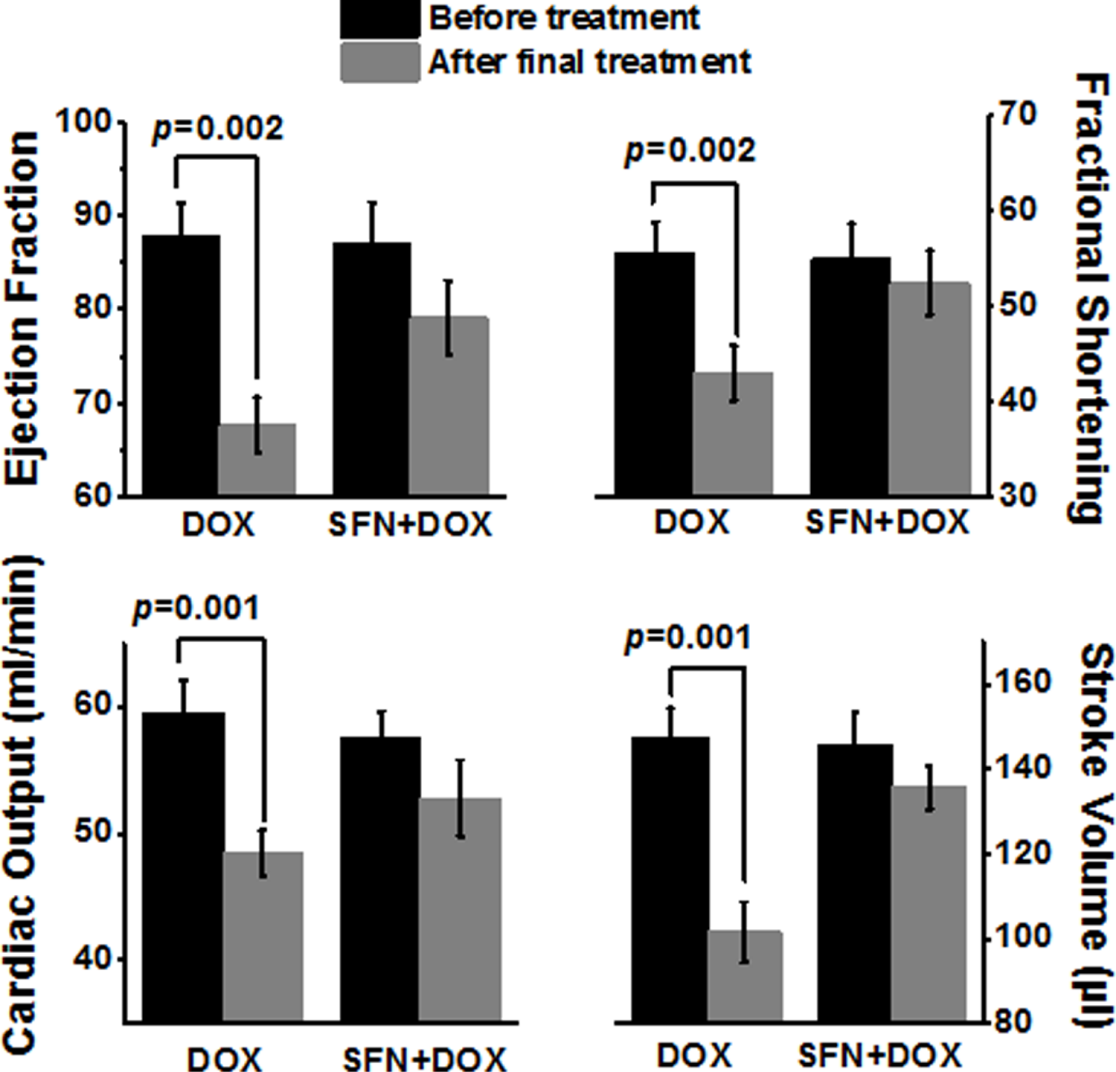
SFN treatment protects rats from DOX-induced cardiomyopathy and preserves cardiac function in non-tumor bearing animals. Adult female Sprague-Dawley rats (n = 8) were treated with i) DOX (total of 20 mg/kg, i.p. for 3 wks), or ii) DOX+SFN (4 mg/kg oral; 5 days/week). Cardiac function (ejection fraction; fractional shortening, cardiac output and stroke volume) was assessed by echocardiography before treatment and the day after completion of treatment.

To determine if SFN provides cardioprotection during chronic DOX treatment, cardiac function was assessed by echocardiography after a full course of DOX treatment in non-tumor bearing rats. Changes in cardiac ejection fraction, fractional shortening, cardiac output and stroke volume in rats treated with DOX alone revealed impaired cardiac function (Fig 2). In contrast, these cardiac functional parameters were essentially normal in the SFN+DOX-treated group.

### SFN improves mitochondrial function and structure during DOX treatment

**Fig 3:**
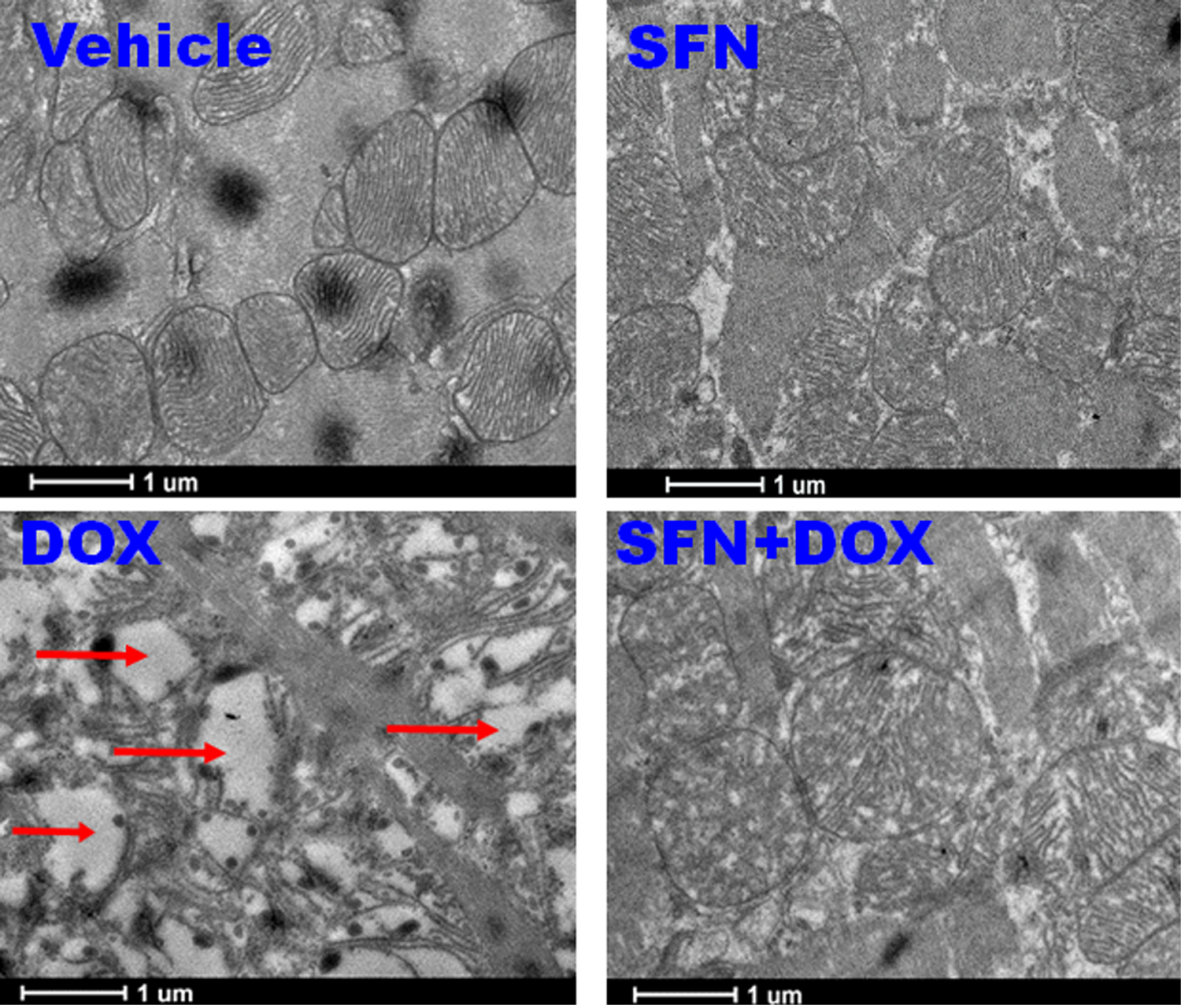
SFN administered during DOX treatment protects the ultrastructure of cardiac mitochondria in non-tumor bearing rats. SFN, in the presence of DOX, prevents mitochondrial cristae disarrangement, partial cristolysis and formation of an electron-lucent matrix. Arrows indicate damaged mitochondria.

DOX metabolism in cardiac cells involves its conversion into a more reactive semiquinone by the mitochondrial complex I of the electron transport chain (ETC), resulting in increased oxidative stress [3, 58]. To determine if SFN could protect mitochondrial function in the heart, ETC activity was measured in left ventricular biopsies by high resolution respirometry. SFN clearly protected the ETC from DOX damage as measured by oxygen flux (Table 1). Moreover, transmission electron microscopy results suggest that SFN protected mitochondria from DOX-induced cristae disarrangement, partial cristolysis and reduced electron density of the matrix (Fig 3). Taken together, these results for the first time demonstrate that SFN protects mitochondria of cardiac cells from DOX toxicity.

**Table 1:** SFN administered during DOX-treatment improves ETC function in non-tumor bearing animals. Respiration status of complex I, II+III, and IV of the ETC was evaluated in fresh left ventricular tissue as described in the Materials and Methods. According to oxygen flux measures (pmol/mg/substrate), co-treatment of rats with SFN+DOX improved complex I, II+III and IV respiration compared to rats treated only with DOX.

	Vehicle	SFN	DOX	SFN+DOX
**Complex I**	48.84±4.37	50.19±1.99	39.34±2.89*	59.60±2.21**
**Complex II&III**	67.07±2.19	72.41±2.71	50.13±1.07*	65.65±3.79**
**Complex IV**	33.21±3.06	37.01±1.87	20.68±1.63*	29.11±1.32**

Mean±SD (n = 8).

*p< 0.0001 DOX *vs* SFN+DOX

**p<0.0002 Vehicle *vs* SFN+DOX.

### SFN reduces DOX-induced oxidative stress in the rat heart

**Fig 4:**
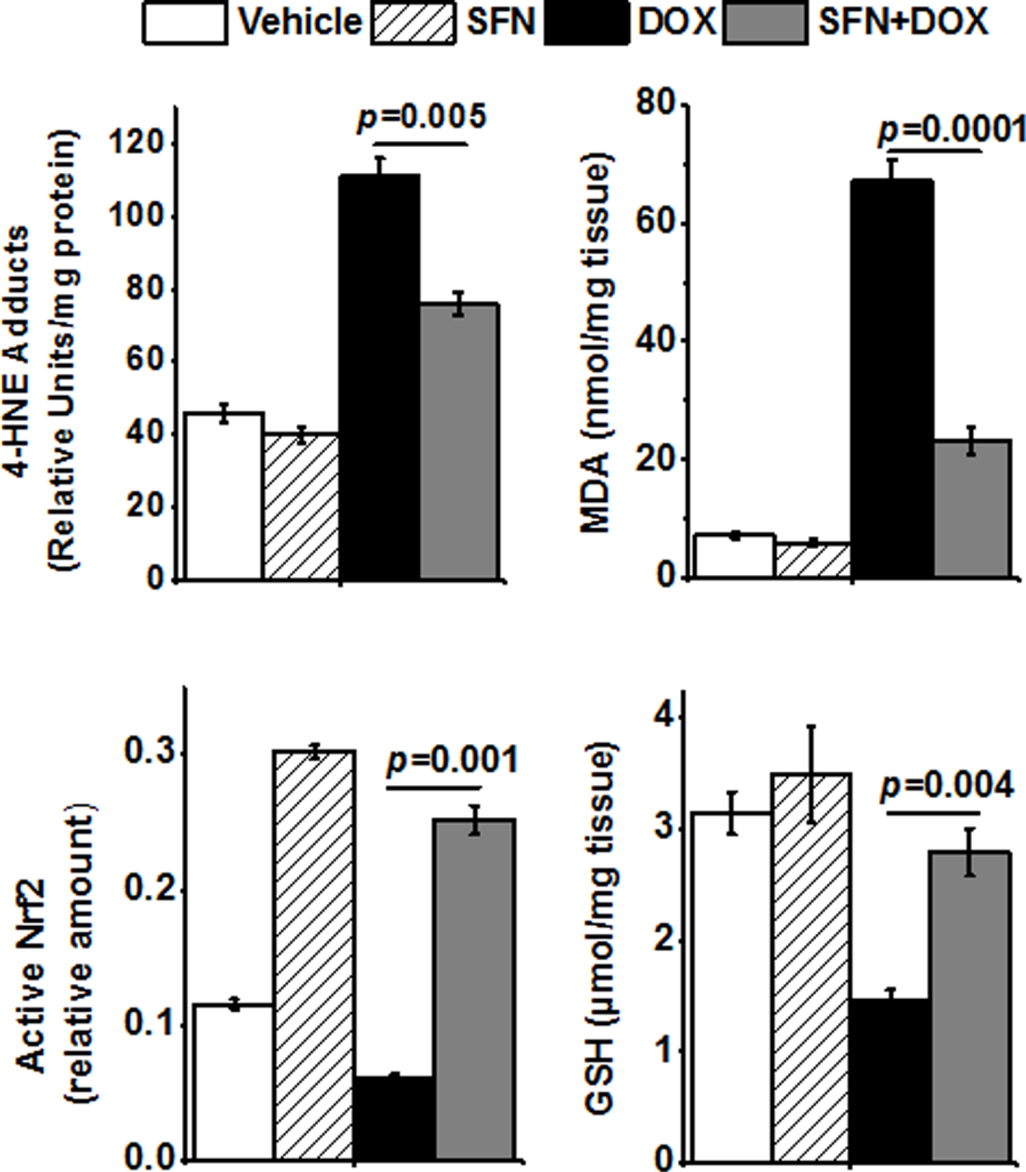
SFN reduces DOX-induced oxidative stress in the heart of non-tumor bearing rats. Isolated cardiac tissue was homogenized and assayed for markers of oxidative stress: 4-HNE adduct levels, MDA content and GSH levels. Nrf2 binding activity was measured by ELISA in nuclear extracts. Data shown are mean±SD of triplicate measurements on homogenates (n = 4).

To determine if SFN co-treatment affected oxidative stress in rat hearts exposed to DOX, we examined three correlates of oxidative stress: increased lipid peroxidation that results in two correlates, generation of MDA and 4-HNE formation, and the depletion of GSH levels. With DOX treatment alone, cardiac MDA and 4-HNE adduct levels were elevated (very significantly for MDA), while GSH was reduced to less than 50% of the level in control hearts. SFN and DOX co-treatment reduced MDA and 4-HNE adduct formation and also prevented DOX-induced depletion of GSH levels (Fig 4).

### SFN preserves key antioxidant and anti-electrophile enzyme activities and activates Nrf2 in rat hearts during DOX treatment

**Fig 5:**
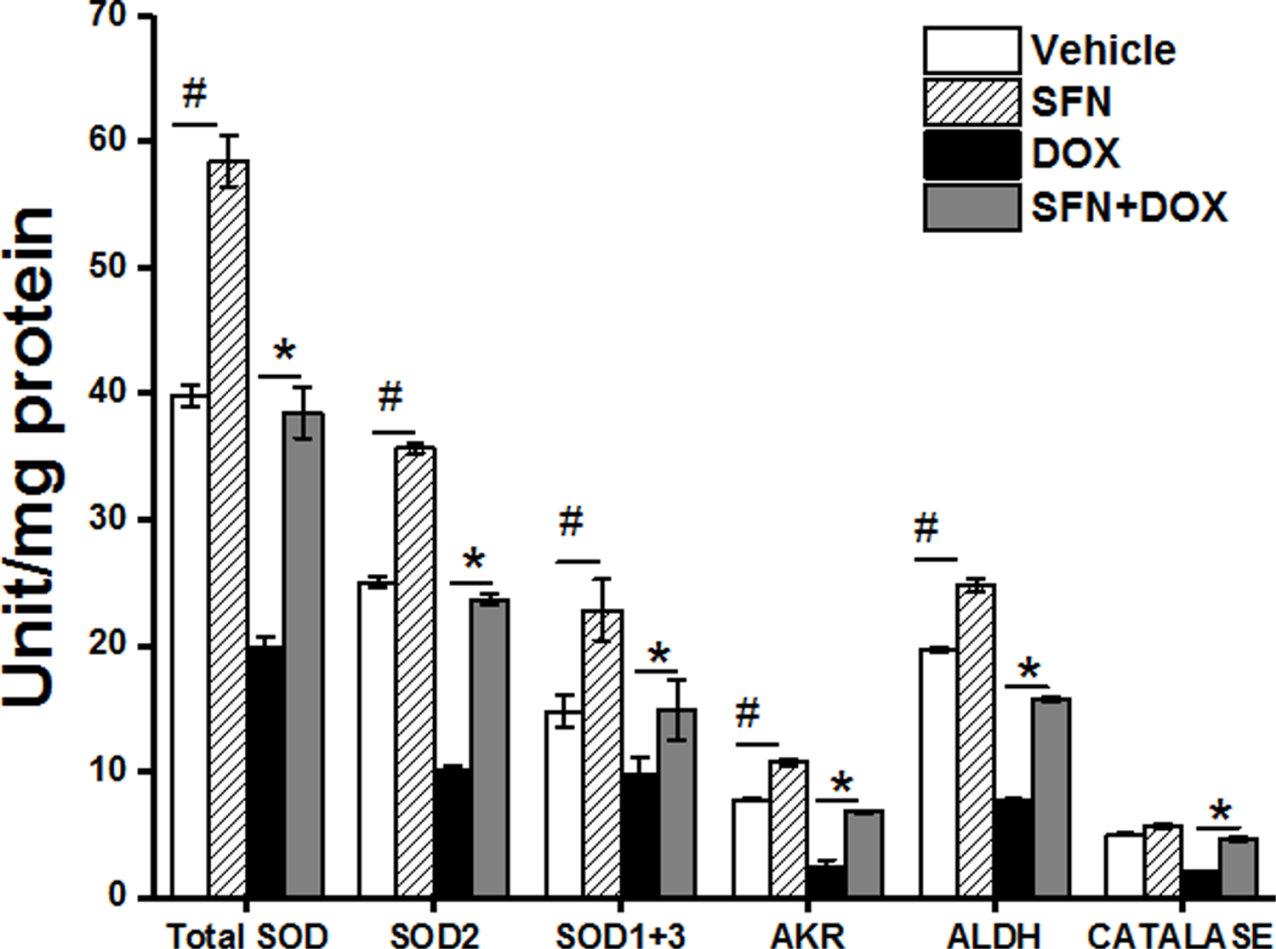
Key antioxidant and anti-electrophile enzyme activities are preserved by SFN in hearts of DOX-treated non-tumor bearing rats. Isolated cardiac tissue from treated animals was homogenized and assayed for SOD, catalase, AKR, and ALDH enzyme activities. Data shown are mean±SD of triplicate measurements on homogenates (n = 4). #*p*<0.001 *vs* vehicle; *p<0.001 *vs* DOX.

To understand a potential primary mechanism by which SFN may reduce cardiac oxidative stress, we examined the levels of active Nrf2 in the rat heart. We demonstrated that SFN strongly activated nuclear Nrf2 in the non-tumor bearing rat heart and even maintained very high Nrf2 activity in the DOX-treated group (Fig 4). We then compared the activities of key antioxidant/anti-electrophile enzymes, encoded by Nrf2 target genes that are primarily active in mitochondria. We found SOD, AKR, ALDH and catalase enzyme activities elevated above control levels in rats treated with SFN alone (Fig 5). In animals co-treated with SFN and DOX, activities of all enzymes were essentially restored to the levels in control animals.

### SFN does not interfere with DOX toxicity or Nrf2 activity in breast cancer cell lines

**Fig 6:**
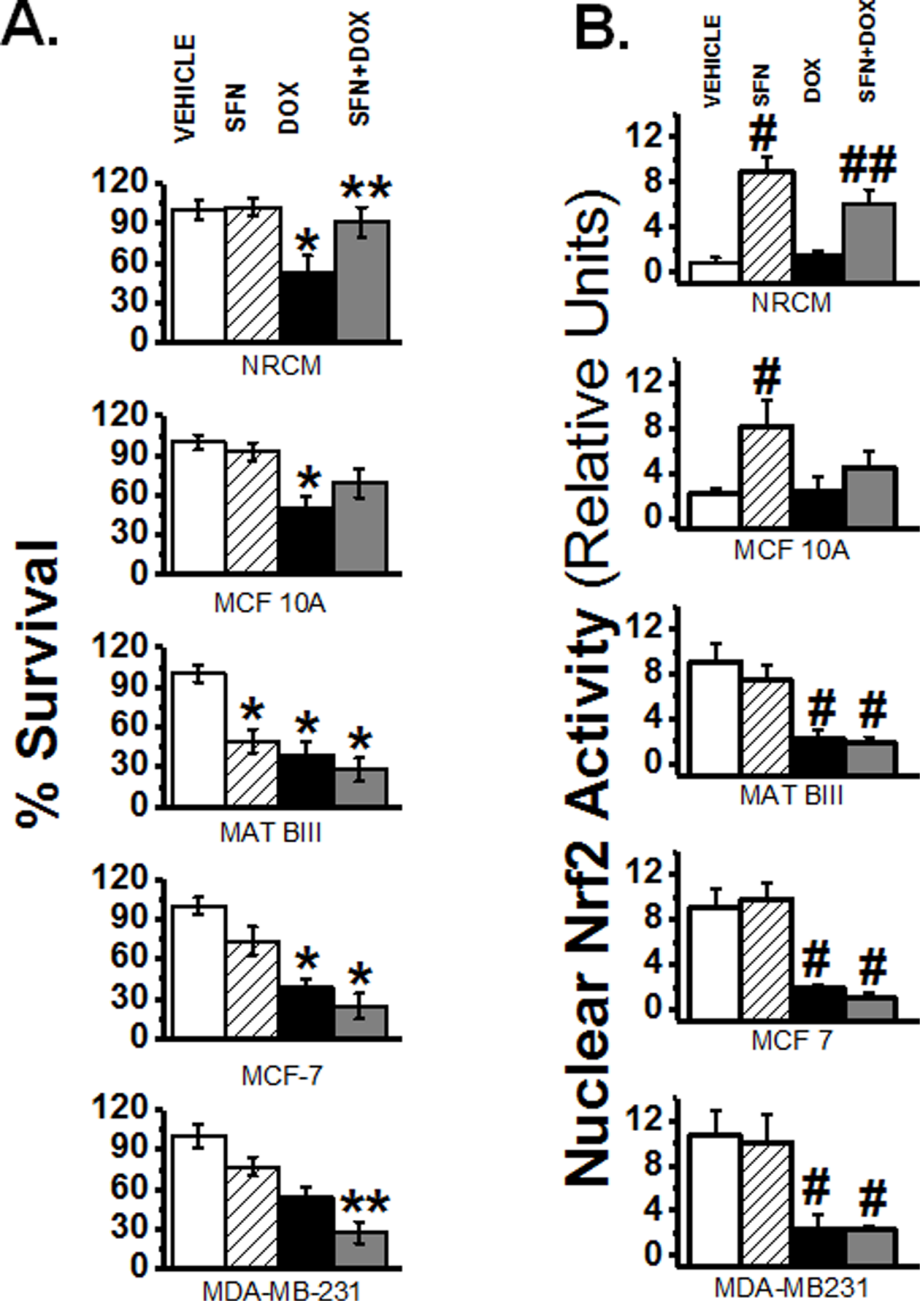
SFN does not interfere with DOX toxicity or Nrf2 activity in breast cancer cell lines. Neonatal rat cardiac ventricular myocytes (NRCM) and four breast cancer cell lines were treated for 24 hrs with either DMSO vehicle, 2.5 µM SFN, 5 µg/ml DOX, or 2.5 µM SFN + 5 µg/ml DOX. **A.** Survival was determined by the calcein blue AM assay (^*^p = 2x10^-4^,^**^p = 2x10^-3^, n = 32). **B.** Active Nrf2 was measured as described in the Materials and Methods (^**#**^*p* = 2x10^-3^,^**##**^*p* = 3x10^-3^_,_ n = 8).

Since SFN+DOX treatment of rats diminished many of the toxic side effects of DOX, SFN might serve as a useful cardioprotectant when using DOX in breast cancer chemotherapy. However, by increasing Nrf2 activity, SFN also upregulates detoxification pathways, which could reduce the efficacy of DOX. Nrf2 deregulation and persistent activation have been associated with chemoresistance in some cancers but not others [59, 60]. To test whether SFN, at concentrations that protect cardiac cells from DOX toxicity, compromise the ability of DOX to kill breast cancer cells, we first assayed survival in cancer and cardiac cells (neo-natal rat cardiomyocyte, NRCM) *in vitro*. As shown in Fig 6A, SFN selectively enhanced sensitivity to DOX in three invasive breast cancer cell types, while reducing DOX sensitivity in the non-invasive MCF 10A line. In contrast, SFN actually promoted the survival of NRCM in the presence of DOX.

We also examined nuclear Nrf2 activity in SFN-, DOX- or DOX+SFN-treated NRCM in comparison to the four cancer cell lines. We detected high, basal levels of nuclear Nrf2 activity in the three invasive cancer cell lines as compared to NRCM and the non-invasive MCF 10A. Furthermore, SFN treatment did not alter Nrf2 activity in the cancer cells, but enhanced the activity in NRCM and MCF 10A cells. This highly induced Nrf2 activity persisted during SFN+DOX co-treatment. Lastly, in the three invasive breast cancer lines Nrf2 activity was not inducible in the presence or absence of DOX (Fig 6B). These results clearly revealed that SFN differentially regulate Nrf2 functions in normal and breast cancer cells.

### SFN acts synergistically with DOX to inhibit HDAC and DNMT activity, decrease ERα detection and increase caspase-3 activity

**Fig 7:**
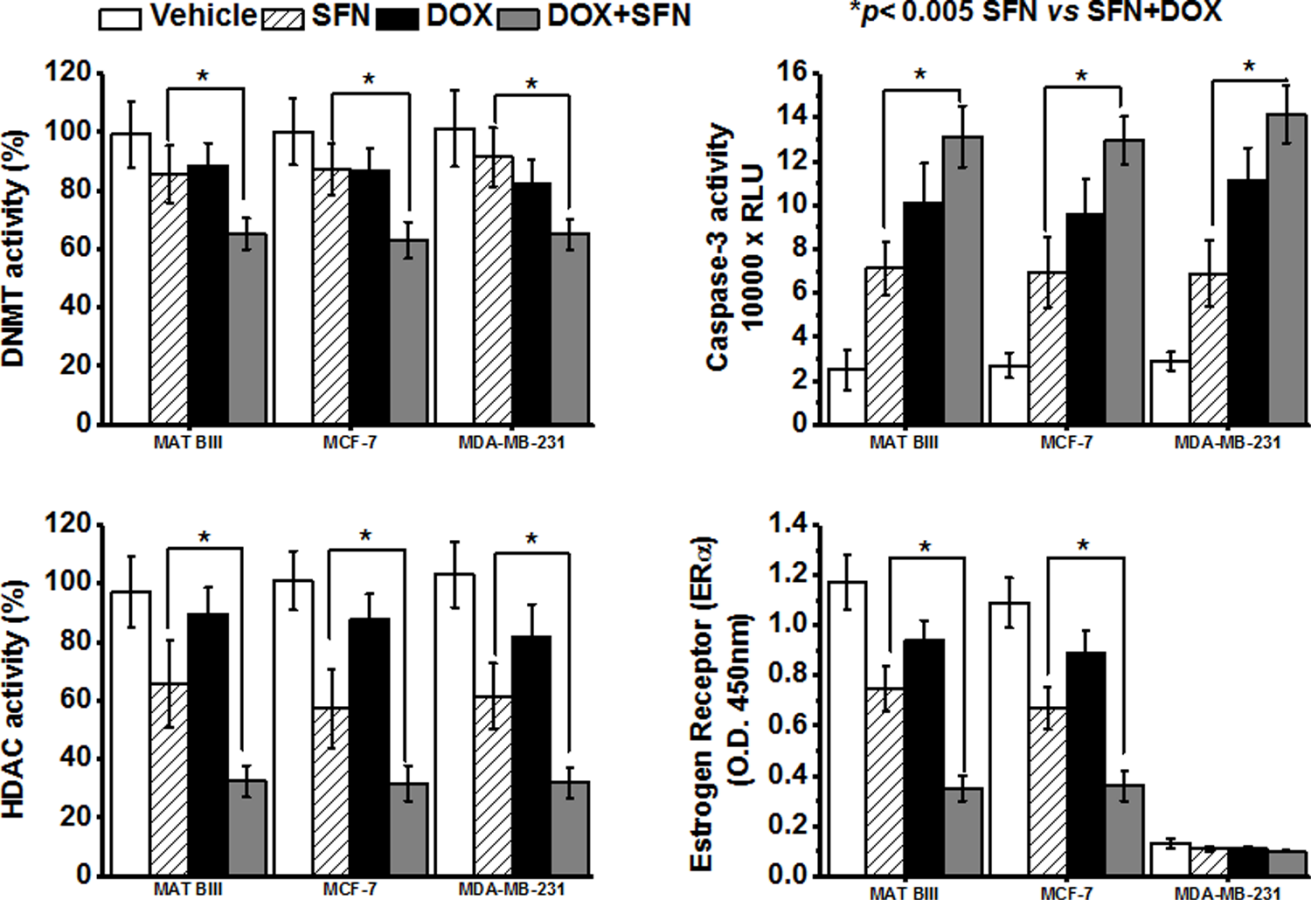
SFN acts synergistically with DOX in inhibition of HDAC and DNMT activity, suppression of ERα and increase in caspase-3 activity in cancer cells *in vitro*. All assays were performed on cancer cells after 48 hrs of DMSO (vehicle/control), 2.5 µM SFN, 5 µg/ml DOX, or 2.5 µM SFN + 5 µg/ml DOX treatment (mean±SD, n = 8).

SFN exhibits anti-cancer activities by activation of caspase-3, inhibition of DNMT and HDAC activity, and by reduction in ERα levels [29–32]; these mechanisms of anticancer activity are different from the mechanism by which DOX kills cancer cells [3, 16, 17]. Hence, we hypothesized that co-treatment with a low dose of SFN could enhance tumor regression by activating other key antitumor mechanisms than DOX. Therefore, we tested the effects of SFN±DOX on the activities of caspase-3, DNMT and HDAC, and on ERα levels in MCF-7, MDA-MB-231 and Mat B III breast cancer cell lines. Our results showed that SFN treatment alone inhibited HDAC activity and reduced ERα levels (except in MDA-MB-231) by 20–40% after 48 hrs in breast cancer cells (Fig 7). This inhibitory effect was further potentiated (40–60%) when we co-treated these cells with SFN and DOX. In addition, the combination of DOX and SFN significantly reduced DNMT activity. Lastly, DOX increased the stimulatory effect of SFN on caspase-3 activity in all three cancer cell lines (Fig 7).

### SFN and DOX co-treatment enhances regression of rat breast cancer tumors

**Fig 8:**
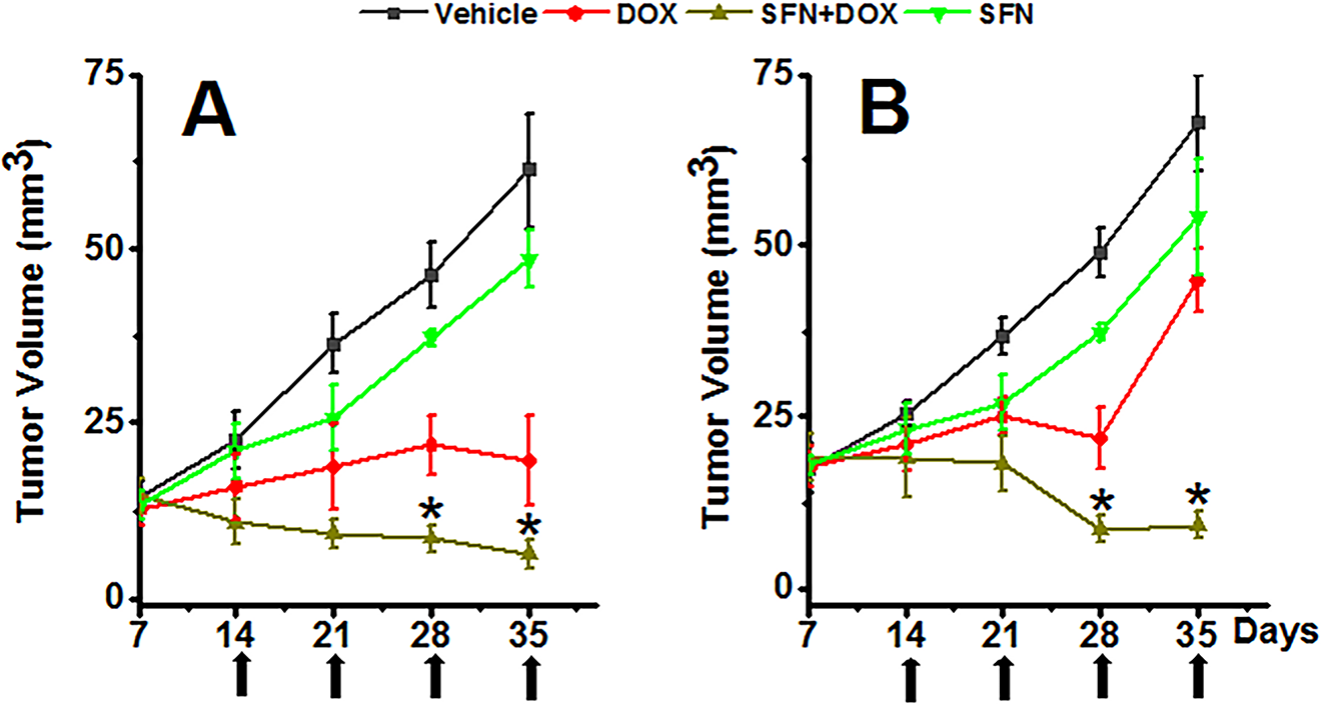
SFN co-treatment enhances tumor regression by DOX in a rat breast cancer model. Effect of metronomic SFN treatment (4 mg/kg, 5 days a week), DOX (A: 5 mg/kg weekly, total 20 mg/kg; B: 2.5 mg/kg weekly, total 10 mg/kg) and SFN+DOX on MAT B III-induced mammary tumors in rats. Data represent the mean±SD of tumor volumes (n = 8). Arrows indicate DOX injections post-tumor inoculation. *p<0.001 vs DOX-treated rats at the same time point.

To test if SFN can interfere with the *in vivo* anticancer activity of DOX, we treated mammary tumor bearing rats with vehicle, SFN, DOX and SFN+DOX and monitored tumor growth. The treatment schedule for this study is shown in Fig 1. SFN alone did not have significant effects on tumor growth, but DOX treatment alone reduced tumor growth relative to the vehicle control. Furthermore, SFN+DOX treatment (with a total DOX dose of 20 mg/kg) was able to eradicate the tumors in all rats by day 35 after tumor implantation (Fig 8A). We also examined SFN+DOX co-treatment at a lower dose of DOX (total 10 mg/kg) and observed significantly better tumor regression with SFN+DOX as compared to SFN or DOX alone (Fig 8B). These results demonstrate that SFN can act synergistically to promote tumor regression, allowing a significant reduction in DOX dose required to treat tumors.

### SFN and DOX co-treatment preserves cardiac function, cardiac Nrf2 activity, and reduces the inflammatory response in tumor bearing rats

**Fig 9:**
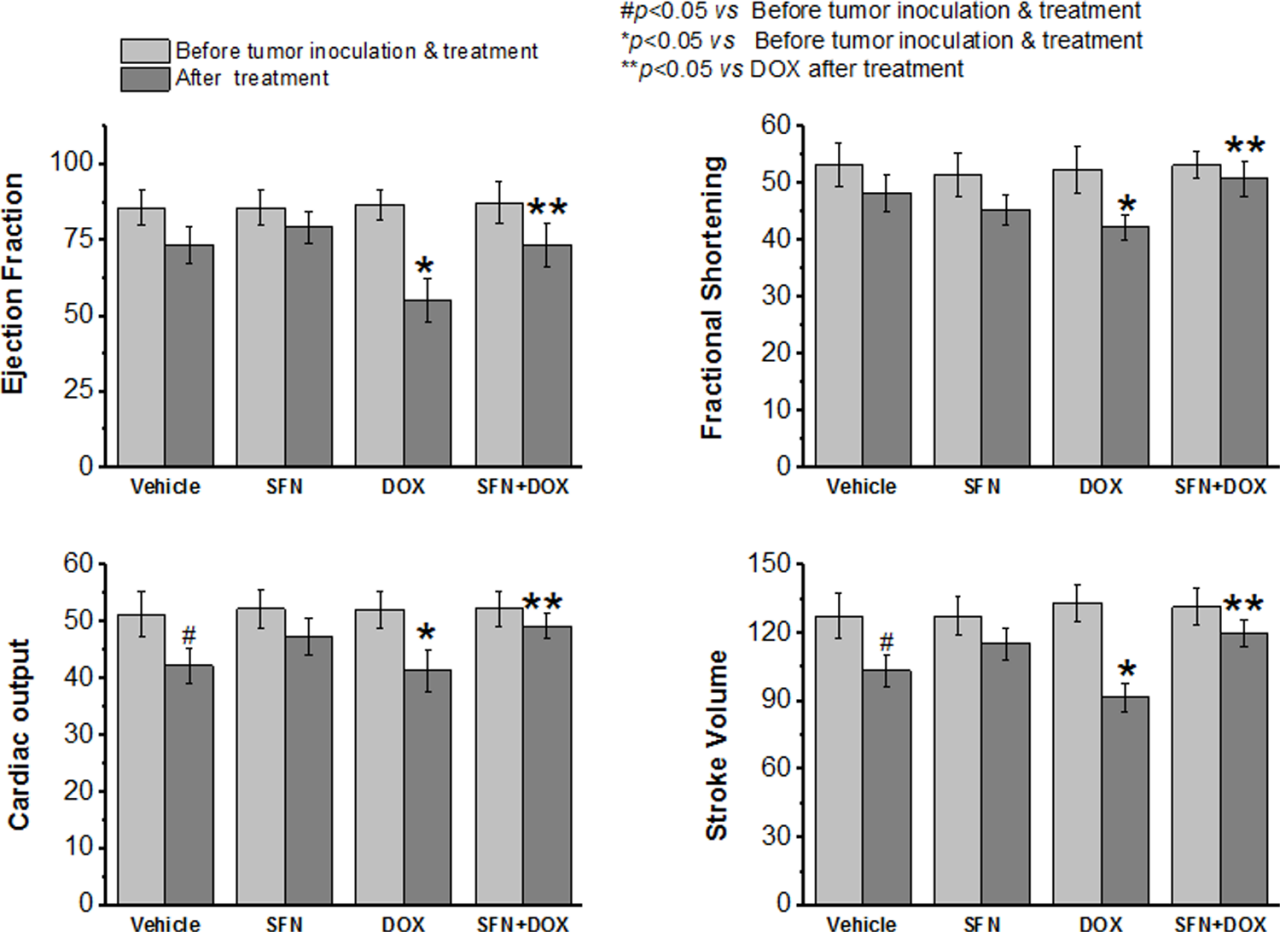
SFN and DOX co-treatment of tumor bearing rats preserves cardiac function. Adult rats were treated with i) DOX (total of 20 mg/kg, i.p. for 4 wks), or ii) DOX+SFN (4 mg/kg oral; 5 days/week). Cardiac function (ejection fraction, fractional shortening, cardiac output and stroke volume) was assessed before tumor inoculation and on the day after completion of treatment (mean±SD, n = 8).

The hearts of cancer patients or small animal models are generally under stress due to the oxidative and inflammatory responses elicited by the cancer [38–42]. Therefore, we examined our rat breast cancer model for cardioprotective actions of SFN during DOX chemotherapy. The presence of the breast tumor significantly affected cardiac output and stroke volume in vehicle-treated groups (Fig 9). While DOX treatment caused the expected reduction in cardiac function, reflected in all four parameters measured on echocardiograms, this effect was significantly attenuated in SFN co-treated rats (Fig 9).

**Fig 10:**
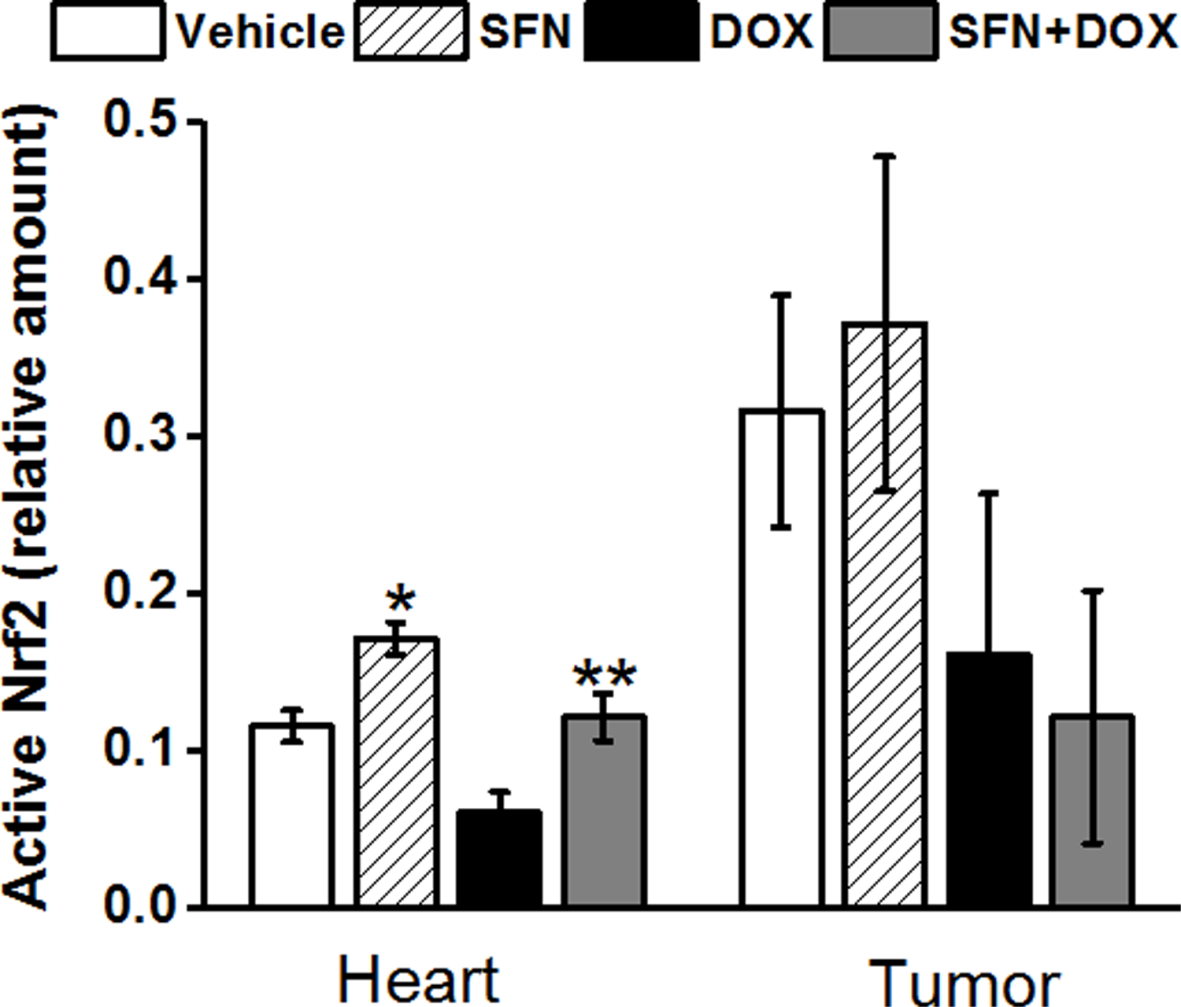
SFN protects Nrf2 activity in hearts of DOX-treated tumor bearing rats. Nrf2 binding activity was measured by ELISA in nuclear extracts from the left ventricle and tumor, isolated after the second DOX injection as described in the Materials and Methods. Data represent the mean±SD of triplicate measurements (n = 5). *p<0.05 SFN vs Vehicle, **p<0.05 SFN+DOX vs DOX treated heart and #*p*<0.05 Vehicle vs DOX.

While in the presence of SFN, cardiac Nrf2 activity was elevated above that of the vehicle control, DOX alone significantly suppressed cardiac Nrf2 activity. However, SFN and DOX co-treatment completely rescued Nrf2 activity in the heart. In contrast, tumor Nrf2 activity was essentially unaffected by the addition of SFN to DOX treatment (Fig 10).

**Fig 11:**
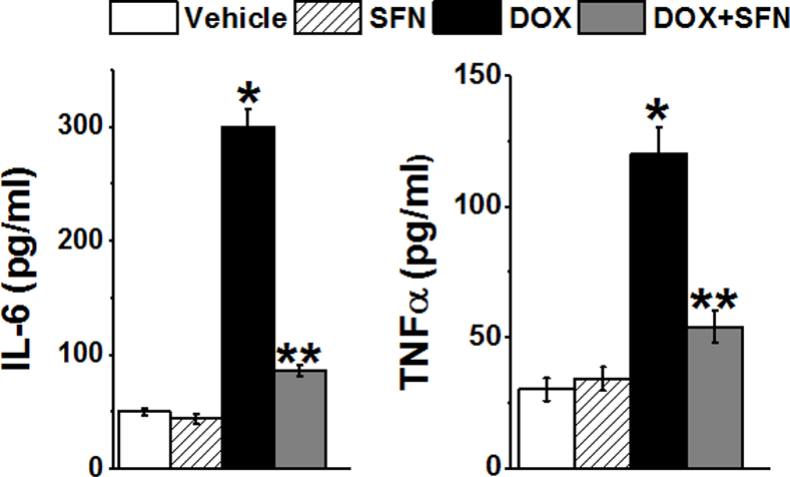
SFN reduces inflammatory cytokine levels in DOX-treated tumor bearing rats. Inflammatory cytokine levels were measured in serum/plasma taken two days after the third DOX injection from rats treated with metronomic SFN±DOX or SFN or Vehicle (PBS). Data represent the mean±SD of triplicate measurements (n = 4). *p< 1x10^-5^ DOX *vs* Vehicle; **p< 2x10^-5^ SFN+DOX *vs* DOX.

To analyze the effect of SFN on DOX-mediated inflammation, we examined the levels of the inflammatory cytokines IL-6 and TNFα in serum. Co-treatment with SFN strongly attenuated DOX-induced serum levels of both cytokines measured two days after the third DOX injection (Fig 11).

## Discussion

Cardiotoxicity often occurs during or after completion of DOX chemotherapy for major malignancies and may predispose to a loss of left ventricular function and congestive heart failure [61–65]. As many as 65% of patients with a childhood malignancy treated with DOX have echocardiographic evidence of left ventricular contractile abnormalities as adults [62]. Despite its frequent occurrence, there are no safe and effective treatments to prevent DOX cardiotoxicity. The opportunity to intervene at the time of DOX chemotherapy to save a patient from acute or delayed cardiac dysfunction is extremely significant and could help in improved quality of life. We used animal and cell culture models to test whether co-treatment with SFN could protect the heart from DOX toxicity but did not interfere with the anti-cancer effects of DOX.

Our first novel finding is that SFN potentiates the effect of DOX on breast tumors. The combination treatment of DOX and SFN was able to eradicate the tumors in all rats by day 35 after tumor implantation. Thus, SFN co-treatment does not impair DOX-induced killing of cancer cells. We further determined whether SFN supplementation lowered the DOX dosage required for effective chemotherapy in the rat breast cancer model. We tested SFN (4 mg/kg oral; 5 days/week for 5 weeks) with DOX (total of 10 or 20 mg/kg i.p. administered over 4 weeks) and showed that in combination with SFN, the dosage of DOX could be lowered by 50% while still eliciting the same anti-cancer effects as DOX alone. Lowering the DOX dosage itself can reduce its side effects not only on the heart but also other normal tissues as well.

SFN is a potent activator of Nrf2, which is a central regulator of cellular responses to electrophilic/oxidative stress. Increased generation of reactive oxygen species (ROS), an altered redox status, and aerobic glycolysis for energy production distinguish highly proliferative cancer cells from normal healthy cells [66–69]. The elevated level of oxidative stress in cancer cells is associated with enhanced use of protective signaling pathways such as those mediated by Nrf2 [70, 71]. Increased Nrf2 activity may help cancer cells evade chemotherapy [72]. Ours and other *in vitro* studies have shown that basal Nrf2 levels in cancer cell lines positively correlate with resistance to DOX [73]. The precise role of Nrf2 in cancer cells, however, is poorly understood [74–77]. Some studies suggest that cancer hijacks the Nrf2-mediated detoxification mechanism for its own progression and to detoxify chemotherapeutic drugs [73, 78, 79]. In contrast, the intrinsic ability of Nrf2 to regulate enzymes that confer defense against oncogenesis characterizes it as an anticancer, chemoprotective protein. Nrf2-KO mouse models display increased sensitivity to carcinogens and toxicants, revealing a tumor-suppressor role of Nrf2 [80]. We showed that Nrf2 activity in cardiac cells, with low Nrf2 activity at baseline, is induced by SFN treatment and, unlike in cancer cells, SFN confers protection from DOX toxicity in cardiac cells. In addition, SFN treatment significantly reduced indications of oxidative stress and increased the activities of Nrf2 target enzymes SOD, CAT, AKR and ALDH in the DOX-treated rat heart. Apparently, SFN enhances the effects of DOX on the tumor, and its mode of action on cancer cells are Nrf2-independent. SFN may exhibit Nrf2-independent anti-cancer activities by enhancing the transcription of tumor suppressor genes, possibly by influencing DNA methylation, activation of caspase-3, induction of cell cycle arrest, and inhibition of ERα, DNMT and TERT [29, 32, 81–86]. Our results indicate that DOX co-treatment with SFN attenuates DNMT and HDAC activity. While this study examined overall activity of these enzymes, further research is required to determine the effects of DOX and SFN on the expression of individual DMNT and HDAC isoforms. Furthermore, SFN lowers ERα levels and SFN+DOX co-treatment enhanced caspase-3 activity. Taken together, these *in vitro* results suggest that SFN+DOX co-treatment modulates epigenetic and pro-apoptotic mechanisms in killing cancer cells.

As DOX and its highly reactive metabolites accumulate in mitochondria, extensive ROS-based stress initiates mitochondrial injury and a vicious cycle of oxidative/electrophilic stress [23, 24]. Results presented in Table 1 and Fig 4 clearly show that SFN indeed restores the mitochondrial functions and integrity altered by the toxic effects of DOX. We showed that SFN mediates protection of mitochondrial function by improved mitochondrial morphology and oxygen flux.

In addition to its direct toxic effects, DOX-induced ROS can induce NF-kB activation, which leads to the expression of inflammatory cytokines such as TNF-α, and IL-6 [12]. SFN-induced activation of Nrf2 inhibits the NF-kB signaling pathway in Dystrophin-deficient *mdx* mice [45]. Results of the present study indicate that SFN lowers serum levels of inflammatory cytokines generated in response to DOX-treatment, demonstrating that SFN plays a major role in reduction of DOX-mediated inflammation.

Various strategies have been designed to attenuate DOX-induced cardiotoxicity, but none of them has proven conclusively helpful. We have determined in rats that SFN, a common and safe dietary supplement, reduces DOX‐linked cardiotoxicity while enhancing the killing of cancer cells by DOX. The translation of this strategy into the clinic would represent a major therapeutic advance in enhancing the efficacy and safety of breast cancer treatment.

## Supporting information

S1 File Excel file containing all pertinent raw data obtained from cell culture and animal experiments in this study. (XLSX)

S2 File PLOS ONE humane endpoints checklist. (DOCX)

S1 Fig SFN treatment protects rats from DOX-induced toxicity and improves survival in non-tumor bearing animals. (TIF)
