# Supplementary figures and images for: Sulforaphane potentiates anticancer effects of doxorubicin and attenuates its cardiotoxicity in a breast cancer model
Source: PLoS One. 2018 Mar 8;13(3):e0193918. doi: 10.1371/journal.pone.0193918 (PMC5843244; doi:10.1371/journal.pone.0193918)

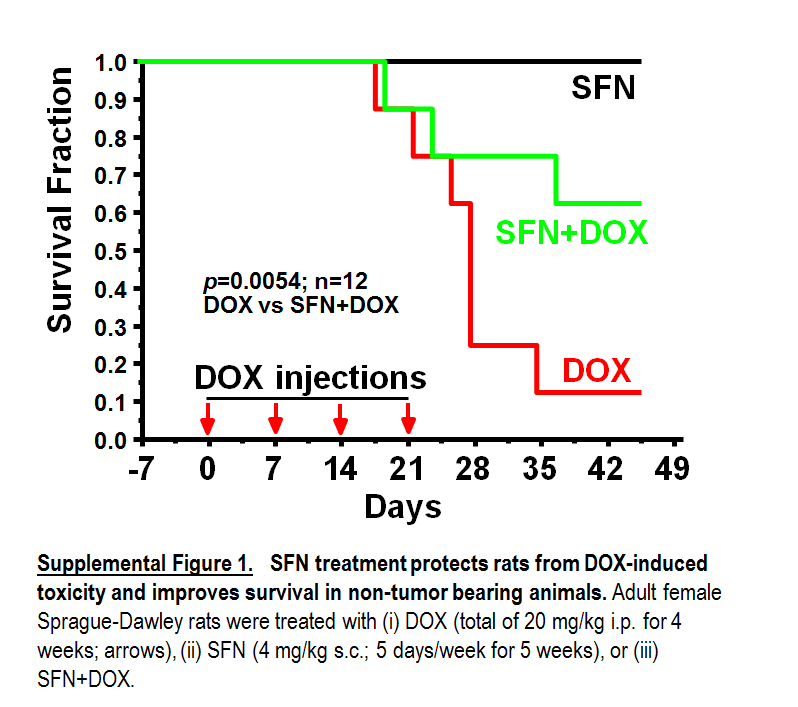

Supplement: S1 Fig — (TIF) [file pone.0193918.s003.tif]
